# WASP-Arp2/3-dependent actin polymerization influences fusogen localization during cell-cell fusion in *Caenorhabditis* *elegans* embryos

**DOI:** 10.1242/bio.026807

**Published:** 2017-07-31

**Authors:** Yan Zhang, Yihong Yang, Zhiwen Zhu, Guangshuo Ou

**Affiliations:** Tsinghua-Peking Center for Life Sciences, School of Life Sciences and MOE Key Laboratory for Protein Science, Tsinghua University, Beijing 100084, China

**Keywords:** Actin polymerization, Arp2/3, Fusogen, Cell-cell fusion, *Caenorhabditis elegans*

## Abstract

Cell-cell fusion is essential for development and physiology. Actin polymerization was implicated in the *Caenorhabditis* *elegans* fusogen EFF-1 engagement in a reconstituted *Drosophila* cell culture system, and the actin-binding protein spectraplakin links EFF-1 to the actin cytoskeleton and promotes cell-cell fusions in *C. elegans* larvae. However, it remains unclear whether and how fusogens and the actin cytoskeleton are coordinated in *C. elegans* embryos. Here, we used live imaging analysis of GFP knock-in and RNAi embryos to study the embryonic cell-cell fusions in *C. elegans*. Our results show that the inhibition of WASP-Arp2/3-dependent actin polymerization delays cell-cell fusions. EFF-1 is primarily distributed in intracellular vesicles in embryonic fusing cells, and we find that the perturbation of actin polymerization reduces the number of EFF-1-postive vesicles. Thus, the actin cytoskeleton differently promotes cell-cell fusion by regulating fusogen localization to the fusing plasma membrane in larvae or to intracellular vesicles in embryos.

## Abstract (precis)

**Summary:** WASP-Arp2/3 regulates fusogen localization to intracellular vesicles in *C. elegans* embryos. Our results indicate that cell-cell fusions rely on distinct mechanisms at different developmental stages.

## INTRODUCTION

Cell-cell fusion is involved in various developmental and physiological events, ranging from sexual reproduction, myogenesis, bone remodeling and immune responses (Chen, 2011; Chen and Olson, 2005; Podbilewicz, 2014). The *Caenorhabditis elegans* fusogenic protein EFF-1 was isolated from genetic screens of epithelial fusion failure and the *eff-1* gene encodes a type I single transmembrane protein (Mohler et al., 2002; Podbilewicz, 2006; Sapir et al., 2007). EFF-1 shares structural homology with the green alga *Chlamydomonas reinhardtii* HAPLESS 2/GENERATIVE CELL SPECIFIC 1 (HAP2/GCS1) family of proteins and viral class II fusion proteins (Fédry et al., 2017; Pérez-Vargas et al., 2014; Valansi et al., 2017; Zeev-Ben-Mordehai et al., 2014), highlighting an evolutionarily conserved origin of somatic cell-cell fusion, fertilization and virus entry.

The actin cytoskeleton regulates myoblast fusion in *Drosophila*, zebrafish and mice (Abmayr and Pavlath, 2012; Chen, 2011; Gruenbaum-Cohen et al., 2012; Kim et al., 2015; Sens et al., 2010; Shilagardi et al., 2013). In a reconstituted *Drosophila* S2R+ cell-cell fusion system, actin polymerization facilitates EFF-1 enrichment and engagement at invasive membrane protrusions (Shilagardi et al., 2013). Our recent results from the *C. elegans* postembryonic cell-cell fusions support the findings from the reconstituted system. In the epithelial seam and hyp7 cell-cell fusions of *C. elegans* larvae, EFF-1 and F-actin accumulate at the cortex of two fusing cells, and WASP-Arp2/3-dependent actin polymerization promotes this process by recruiting EFF-1 to the fusing plasma membranes (Yang et al., 2017). Importantly, we identified that the actin-binding protein spectraplakin/VAB-10A directly links EFF-1 to the actin cytoskeleton (Yang et al., 2017).

However, in *C. elegans* embryos, RNA interference of the actin nucleation factor Arp2/3 complex and the actin nucleation-promoting WAVE/Scar complex did not perturb embryonic epidermal cell fusion (Patel et al., 2008; Xiong et al., 2011). Using an EFF-1::GFP knock-in nematode, we showed that EFF-1 is primarily distributed to intracellular vesicles and may only transiently localize at fusion sites in embryonic fusing cells, which is consistent with the EFF-1 localization pattern uncovered using immunofluorescence and a functional GFP reporter (Smurova and Podbilewicz, 2016). These results indicate that the actin cytoskeleton may be dispensable for intercellular fusion in embryos, raising the question of whether different types of cell-cell fusion use distinct mechanisms for fusogen recruitment and engagement. Hence, we performed fluorescence time-lapse analysis of cell-cell fusions in RNAi-treated embryos, showing that WASP-Arp2/3-dependent actin polymerization is involved in embryonic hyp7 cell-cell fusions by recruiting EFF-1 to intracellular vesicles. Our results indicate that cell-cell fusions rely on distinct mechanisms at different developmental stages in the formation of a single epithelium syncytium.

## RESULTS AND DISCUSSION

### WASP-Arp2/3-dependent actin polymerization promotes embryonic cell-cell fusions

**Fig. 1.**
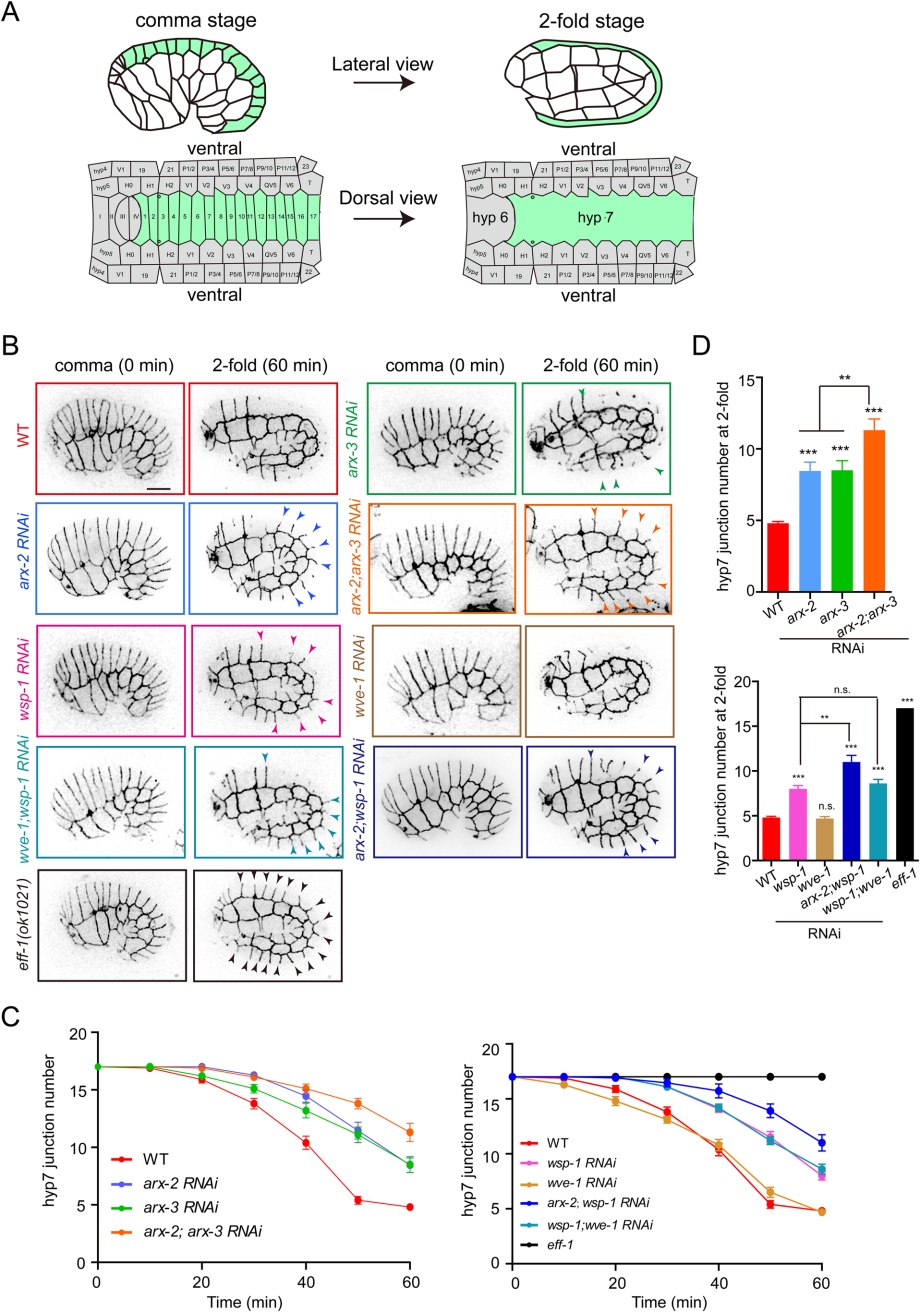
**The WASP-Arp2/3 promotes cell-cell fusion in *C. elegans* embryos.** (A) Schematics of the embryonic epithelial cell fusion from the comma stage (395 min after the first cleavage, left) to twofold stage (450 min, right). Dorsal hypodermal cells 1-17 (green) and several ventral cells fuse to form the main body epithelial syncytium of hyp7. Based on Podbilewicz and White (1994). (B) Inverted fluorescence time-lapse images of the dorsal hypodermal cell fusions from comma (0 min) to twofold stage in WT, RNAi embryos or *eff-1(ok1021)* embryos. Cell boundaries were labeled with DLG-1::TagRFP. Arrowheads indicate the hyp7 precursor cell borders, and 15 borders exist in the comma stage. Scale bar: 10 μm. See Movie 1 for the entire series. (C) Quantification of the dorsal hypodermal cell number from comma to twofold stage; *n*=15-20 for each measurement. (D) Quantification of the dorsal hypodermal cell number at twofold stage. Data are mean±s.e.m.; **P<0.01, ***P<0.001 based on Student's *t*-test; *n*=15-20 for each measurement.

**Fig. 2.**
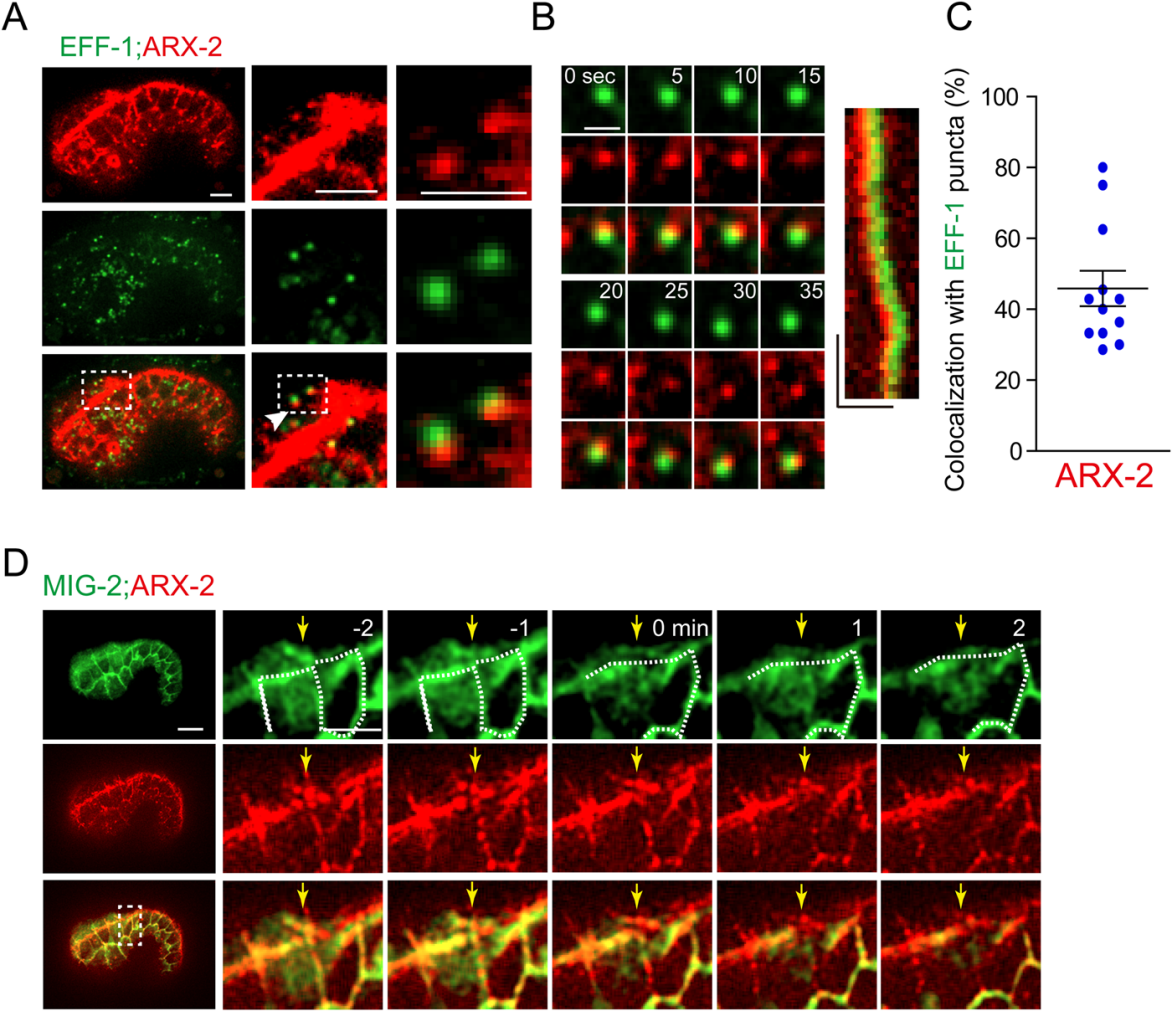
**ARX-2 partially associates with EFF-1 during embryonic cell-cell fusions.** (A) EFF-1::GFP puncta associate with ARX-2::TagRFP puncta in the two fusing hyp7 precursor cells. Areas in the rectangles are enlarged on the right. Scale bars: 5 μm. See Movie 2. (B) Fluorescence time-lapse images of EFF-1 and ARX-2 puncta (from A). Scale bar: 2 μm. Kymograph (right) of EFF-1::GFP and ARX::TagRFP motility. Horizontal bar, 2 μm; vertical bar, 10 s. *n*>5. (C) Ratio of EFF-1 puncta (>0.25 μm^2^) that colocalize with ARX-2 puncta. The number of EFF-1 puncta that colocalized with ARX-2 puncta was divided by the total number of EFF-1 puncta. Data are mean±s.e.m.; *n*=12. (D) Fluorescence time-lapse images of ARX-2::TagRFP knock-in and the plasma membrane (GFP::MIG-2). The right panels show the magnified region of the inset area. Arrows indicate disassembly of the plasma membrane. 0 min, fusion pore formation. Scale bars: 5 μm. See Movie 3 for the entire series.

**Fig. 3.**
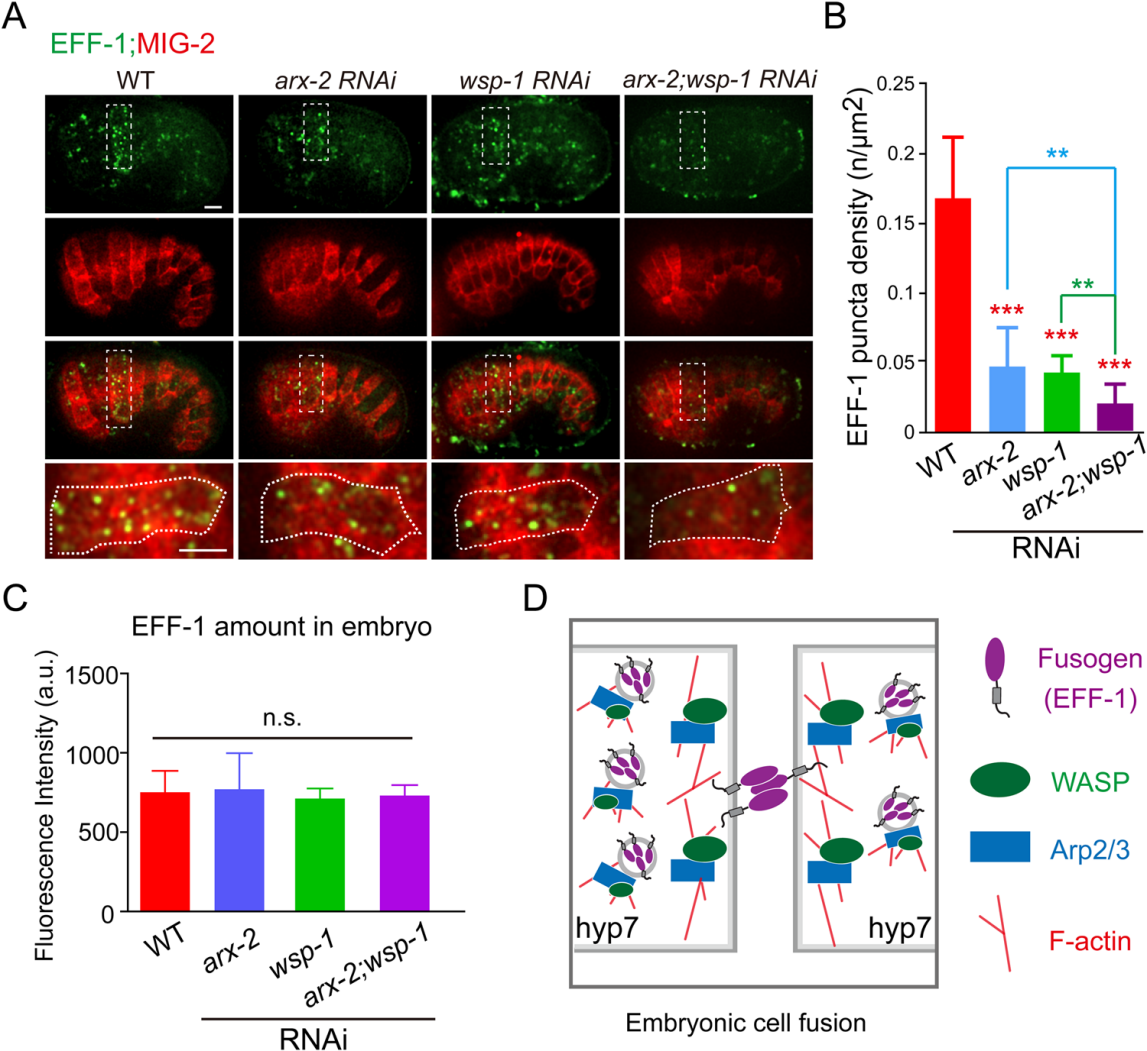
**WASP and Arp2/3 regulate vesicular localization of EFF-1.** (A) Live images of EFF-1::GFP and the plasma membrane (labeled with TagRFP::MIG-2) in WT and RNAi embryos. Each bottom panel represents the magnified region of the top inset area with 90° rotation. The dorsal hypodermal hyp7 cell is outlined with a dotted line. Scale bars: 5 μm. (B) The density of EFF-1 puncta (>0.25 μm^2^) in hyp7 precursor cells of WT and RNAi embryos. Data are mean±s.d. **P<0.01, ***P<0.001 based on Student's *t-*test; *n*=10. (C) Quantification of the amount fluorescence intensity of EFF-1::GFP in embryos. Data are mean±s.d.; no significance based on Student's *t-*test; *n*=15. (D) Proposed models of the coordination between F-actin and EFF-1 during the *C. elegans* embryonic hyp7 precursor cell fusion.

The largest *C. elegans* epithelial hyp7 syncytium contains 139 nuclei, which is formed when embryonic cell-cell fusion merges 23 cells and the remaining 116 cells fuse in larvae (Podbilewicz and White, 1994; Sulston and Horvitz, 1977). To understand whether the actin cytoskeleton plays a general role in *C. elegans* cell-cell fusion, we examined the dorsal hyp7 cell fusion in Arp2/3 and WASP RNAi embryos. The previous RNAi of the ARX-2 subunit in the Arp2/3 complex did not perturb hyp7 cell fusion, and the animals could survive for >3 days (Patel et al., 2008). Our RNAi treatment caused 100% animal lethality within 2 days (*n*>200 for each genotype of WASP, Arp2/3 or WAVE), which is suggestive of a more potent depletion. To visualize the cell boundary of two fusing epithelial cells, we constructed a knock-in animal of TagRFP-tagged DLG-1 that is orthologous to the *Drosophila* Discs large and localizes at apical adherens junctions in all epithelia (Fig. 1B) (Smurova and Podbilewicz, 2016). We also used GFP or TagRFP-tagged small GTPase MIG-2 to visualize the plasma membrane (Fig. 2D and Fig. 3A) (Ou and Vale, 2009). We performed fluorescence time-lapse imaging analysis of embryonic cell-cell fusions. A total of 17 dorsal hyp7 precursor cells merged their plasma membranes to form the hyp7 syncytium (Fig. 1A). The loss of DLG-1::TagRFP fluorescence at the border of two hyp7 cells indicates the completion of cell-cell fusion (Fig. 1B).

In DLG-1::TagRFP embryos, the red fluorescent cell boundary between two fusing hyp7 cells progressively disappeared (Fig. 1B). At the twofold developmental stage, only 4±1 (*n*=12) borders could be detected, indicative of the completion of 13±1 hyp7 cell-cell fusions. However, all the DLG-1::TagRFP fluorescence was retained in *eff-1(ok1021)* mutant embryos at the same development stage (Fig. 1B). RNAi treatment of *arx-2*, *arx-3* or *wsp-1* delayed 9±2, 8±2 or 9±1 hyp7 precursor cell fusions at the twofold stage, respectively (*n*=10-12; Fig. 1B-D; Movie 1). The delayed cell-cell fusions in WASP and Arp2/3 RNAi embryos may result from an incomplete depletion of WASP or Arp2/3 using RNAi. Importantly, 11±3 and 11±2 hyp7 precursor cells (*n*=10-12) did not fuse in *arx-2;arx-3* and *arx-2;wsp-1* double RNAi embryos at this stage, indicating an enhancement of cell fusion defects (Fig. 1B-D). Consistent with this notion, the larval cell-cell fusion defects were also enhanced in *wsp-1* and *arx-2* double conditional knockouts, presumably because of the additive disruption of WASP and Arp2/3 (Yang et al., 2017). In agreement with the previous data, RNAi of *wve-1* did not cause any apparent defects of cell-cell fusion in embryos (Fig. 1B-D), which indicates that WASP, but not WAVE, is the essential actin nucleation promoting factor during cell-cell fusions. Thus, the WASP-Arp2/3-mediated actin polymerization is involved in cell-cell fusion in *C. elegans* embryos.

### The actin cytoskeleton regulates the vesicular localization of EFF-1 in embryos

To further investigate the coordination between EFF-1 and actin polymerization in embryos, we generated EFF-1::GFP and ARX-2::TagRFP double knock-in animals. We performed studies by quantifying the percentage of endogenous EFF-1::GFP puncta that were associated with ARX-2::TagRFP puncta in hyp7 precursor cells (Fig. 2A-C; Movie 2). Forty-five percent of EFF-1 puncta overlapped with ARX-2 puncta (Fig. 2C). Moreover, our kymograph analysis revealed that EFF-1::GFP and ARX-2::TagRFP move together in some migratory puncta (Fig. 2B). To examine whether actin polymerization is required for EFF-1::GFP distribution in hyp7 precursor cells, we compared the EFF-1::GFP puncta density in WT or *arx-2* and *wsp-1* RNAi embryos. WT embryos contained 0.17±0.04 EFF-1::GFP puncta per µm^2^, whereas RNAi of *arx-2*, *wsp-1* or both reduced the puncta density to 0.05±0.02, 0.04±0.01 and 0.02±0.01 per µm^2^, respectively (Fig. 3A,B). When we quantified the overall levels of EFF-1::GFP, we did not observe any statistically significant changes in the fusing cells of *arx-2* or *wsp-1* RNAi embryos (Fig. 3C). These results indicate that WASP and Arp2/3 do not affect the expression or the stability of EFF-1, but regulate EFF-1 vesicular localization, suggesting that EFF-1 may be evenly distributed to the cytoplasm in *C. elegans arx-2* or *wsp-1* embryos.

Considering that EFF-1::GFP forms puncta in the postembryonic seam V.a (anterior daughter cell of the V cell) cells, we examined whether WASP is also involved in the membrane trafficking of EFF-1 in larval seam cells (Yang et al., 2017). Using the same protocol as for EFF-1 quantification in embryos, we found that the WT V.a cells and *wsp-1*-deficient cells contained 0.14±0.03 and 0.12±0.02 EFF-1::GFP puncta per µm^2^, respectively (mean±s.d., *n*=16, no statistical significance based on Student's *t-*test). Although the transmembrane protein EFF-1 must be synthesized in the endoplasmic reticulum and undergo membrane trafficking to arrive on the plasma membrane in both embryonic and larval cells, our results indicate that the underlying regulatory mechanisms appear to be different at distinct developmental stages (Fig. 3D) (Yang et al., 2017).

EFF-1 has distinct localization patterns at different developmental stages in forming a single epithelial syncytium. Although the actin cytoskeleton is generally involved in this process, it facilitates EFF-1 localization in a distinct manner in embryos and larvae. These results indicate that cell-cell fusion may be more complex than is currently appreciated. A previous study revealed that EFF-1 is removed from the plasma membrane by RAB-5 and dynamin-mediated endocytosis in embryos (Smurova and Podbilewicz, 2016). We showed that ARX-2 puncta always localized at the borders of the fusing hyp7 cells (Fig. 2D, *n*>50 for each marker; Movie 3) and that EFF-1 puncta could occasionally be detected at the border (Yang et al., 2017). Because WASP and Arp2/3 are essential for membrane endocytosis (Firat-Karalar and Welch, 2011), the reduction of EFF-1 puncta in the absence of WASP or Arp2/3 can be explained by defective endocytosis. However, the perturbation of endocytosis led to the hyperfusion phenotype (Smurova and Podbilewicz, 2016) rather than the delay of cell-cell fusion observed in WASP or Arp2/3 RNAi embryos. Given that Arp2/3-based actin nucleation is also required for ER-to-Golgi transport (Campellone et al., 2008), and that EFF-1 puncta colocalize with the Golgi (Smurova and Podbilewicz, 2016), Arp2/3-dependent secretory sorting may facilitate the trafficking of EFF-1 from the ER to the plasma membrane, thereby promoting cell-cell fusion.

## MATERIALS AND METHODS

### *C. elegans* strains, genetics and DNA manipulations

Strains were maintained on nematode growth medium (NGM) plates seeded with *Escherichia coli* OP50 following standard protocols at 20°C. CRISPR-Cas9-assisted knock-in animals were generated and analyzed as described previously (Dickinson et al., 2013; Shen et al., 2014). To construct knock-in repair template plasmids, we amplified the 1-1.5 kb upstream and downstream homologous arms from the N2 genomic DNA and inserted them into *pPD95.77* with an In-Fusion Advantage PCR Cloning Kit (Clontech, Mountain View, USA). To avoid the cleavage of the homologous repair template by Cas9, synonymous mutations were introduced to the Cas9 target site of the template. The sgRNA plasmid and the knock-in repair template plasmid were co-injected into N2 animals. The knock-in worms were selected and examined by PCR and Sanger sequencing. Transgenic worms were generated by microinjection of DNA plasmids to the germline at 10-50 ng/µl with the co-injection markers *pRF4* [*rol-6 (su1006)*] and *Podr-1::dsRed* into N2 animals. The primers, plasmids, PCR products and strains are listed in Tables S1-S4.

### Feeding RNAi

Feeding RNAi was performed as described previously (Kamath et al., 2001). For *arx-2*, *arx-3*, *arx-5*, *arx-6*, *arx-7* and *wsp-1*, the clones from the Ahringer RNAi feeding library were used (Kamath and Ahringer, 2003). Bacterial cultures for the targeted gene were grown for 8-12 h on plates containing NGM agar+1 mM IPTG+25 μg/ml carbenicillin. L4 young adult worms were fed at 20°C for 16-24 h. F1 embryos were collected and analyzed.

### dsRNA preparation and microinjection

Double-stranded *wve-1 RNA* was made by *in vitro* transcription with a T7 RNA RiboMAX Express RNAi System Kit (Promega, Madison, USA). dsRNA was annealed by heating to 90°C for 2 min and cooling by 1° every 8 s until reaching 25°C. dsRNA injections used 1 mg/ml dsRNA in water and were injected to DLG-1::TagRFP knock-in strains.

### Live-cell imaging in *C. elegans* embryo

*C. elegans* eggs in M9 buffer were mounted on 3% agarose pads at 20°C (Chai et al., 2012). Live-cell images were collected with an Axio Observer Z1 microscope (Carl Zeiss MicroImaging, Jena, Germany) equipped with a 100×/1.45 NA objective or a IX83 microscope (Olympus, Southend-on-Sea, UK) equipped with a 150×/1.45 NA oil objective, an EM CCD camera (Andor iXon+DU-897D-C00-#BV-500, Andor Technology, Belfast, UK), and the 488 nm and 568 nm lines of a Sapphire CW CDRH USB Laser System (Coherent, Santa Clara, USA) with a spinning disk confocal scan head (CSU-X1 Spinning Disk Unit, Yokogawa, Kanazawa, Japan). Time-lapse images were acquired with an exposure time of 200 ms every 2 min for imaging the entire cell-cell fusion process. Images were acquired with μManager software (https://www.micro-manager.org/), and processed and quantified with ImageJ software (http://rsbweb.nih.gov/ij/).

### Quantification and statistical analysis

To quantify the dorsal hypodermal cell fusions in embryos, we measured the number of the dorsal hyp7 cell borders marked by DLG-1::TagRFP every 10 min from comma stage to twofold stage. EFF-1 puncta (>3 pixel size) density in embryos was measured from the dorsal hypodermal cell 3. To quantify the EFF-1 amount in embryos, we measured the fluorescence intensity of all dorsal hypodermal 7 precursor cells. We used the Student's *t*-test to determine significant differences in cell-cell fusion between WT and mutants, as indicated in the figure legends.
